# Clinical Utility of Opportunistic Genome-Wide cfDNA Prenatal Screening in Intermediate-Risk Pregnancies

**DOI:** 10.3390/genes16111344

**Published:** 2025-11-07

**Authors:** S. Menao Guillén, L. Pedrola, C. Orellana, M. Roselló, M. Arruebo, C. Lahuerta Pueyo, M. Sobreviela Laserrada, B. Marcos, J. Pascual Mancho, J. V. Cervera, M. Tajada, R. Quiroga

**Affiliations:** 1 Department of Genetics, Hospital Clínico Universitario Lozano Blesa, 50009 Zaragoza, Spain; 2 Department of Genetics, Hospital Universitario La Fe, 46026 Valencia, Spain; pedrola_lai@gva.es (L.P.); orellana_car@gva.es (C.O.); rosello_mpi@gva.es (M.R.); cervera_jos@gva.es (J.V.C.); 3 Department of Genetics, Hospital Universitario Miguel Servet, 50009 Zaragoza, Spain; marruebo@salud.aragon.es (M.A.); clahuertap@salud.aragon.es (C.L.P.); 4 Obstetrics Department, Gynecological Center Bolonia, 50009 Zaragoza, Spain; mercedessobreviela@gmail.com; 5 Obstetrics Department, Hospital Universitario La Fe, 46026 Valencia, Spain; marcos_bea@gva.es (B.M.); quiroga_ram@gva.es (R.Q.); 6 Obstetrics Department, Hospital Universitario Miguel Servet, 50009 Zaragoza, Spain; jpascualm@salud.aragon.es (J.P.M.); mctajada@salud.aragon.es (M.T.)

**Keywords:** prenatal cfDNA screening, genome-wide, rare autosomal aneuploidies, copy number variations, trisomy

## Abstract

Background: Non-invasive prenatal testing (NIPT) based on cell-free fetal DNA (cfDNA) in maternal blood has revolutionized prenatal screening for trisomies 21, 18, and 13. This approach, based on next-generation sequencing (NGS), usually allows the detection of other chromosomal abnormalities; however, their clinical value in routine practice requires further evidence. Objectives: This study aimed to assess the experience and clinical utility of genome-wide NIPT in pregnant women at intermediate risk in the autonomous communities of Aragón and Valencia, Spain. Methods: For this purpose, a retrospective cohort study was conducted between 2020 and 2024 across two public hospitals. Pregnant women at intermediate risk for trisomies 21, 18, or 13, were included, as well as those meeting specific clinical criteria. Participants were offered either basic or expanded NIPT, and positive results were confirmed by invasive prenatal testing or placental analysis. Results: Among 9,059 expanded NIPT tests, 132 (1.45%) indicated a high-risk result for less common chromosomal anomalies, comprising 60 rare autosomal aneuploidies (RAAs), 39 copy number variants (CNVs), 23 sex chromosome aneuploidies (SCAs), and 10 multiple abnormalities. The positive predictive value (PPV) was 5.5% for RAAs in the fetus, 12.8% for CNVs (31% for deletions), and 58% for SCAs. Conclusions: Several confirmed anomalies were clinically significant and would not have been detected through conventional screening. Opportunistic use of expanded NIPT enables the detection of additional clinically relevant abnormalities, potentially improving obstetric management without substantially increasing invasive testing.

## 1. Introduction

The identification of cell-free fetal DNA (cfDNA) in maternal plasma by Lo et al. in 1997 marked a breakthrough in the field of prenatal diagnosis [1]. This discovery enabled the development of non-invasive prenatal testing (NIPT), a technique that analyzes placental-derived cfDNA in maternal blood.

Since its introduction, NIPT has become a widely adopted tool that has transformed prenatal screening strategies worldwide [2]. Its progressive incorporation into clinical practice over the past decade is due to its high sensitivity and specificity compared with traditional methods, particularly in the detection of the most common trisomies (21, 18, and 13) [3,4]. In line with this evolution, the American College of Medical Genetics and Genomics (ACMG) has endorsed its use as a first-line screening test for common trisomies in both singleton and twin pregnancies, as well as for sex chromosome aneuploidies (SCAs) in singleton gestations, emphasizing its applicability in the general population beyond high-risk groups [5].

From a methodological perspective, this approach can be extended not only to the most frequent trisomies (chromosomes 21, 18, and 13) but also to the rest of the chromosomes, and even to large copy number variants (CNVs), with its scope depending on the methodological option used. In contrast to the common trisomies—where NIPT has demonstrated high sensitivity along with strong positive and negative predictive values—the reliability data for other chromosomal abnormalities remains under evaluation. Multiple studies and meta-analyses have addressed this issue [6,7,8,9,10,11].

While some studies use NIPT as a universal first-line screening, we used NIPT as a second-line test for pregnant women at intermediate risk based mainly on the combined first trimester test. This approach limits direct comparison with studies involving low-risk pregnancies. Recent reports have documented clinical experience with this expanded strategy, with some identifying additional relevant findings that would not have been detected through traditional screening. Overall, patients with a positive result, involving less-common anomalies, are at higher risk for adverse perinatal outcomes than those with a negative result, though the risk varies based on the specific anomaly detected, implicated chromosome, imprinting status, mosaicism percentage, and other factors. Furthermore, the degree of intervention that can be undertaken in patients with a positive result is not clearly defined, which raises questions regarding its clinical utility.

In this retrospective cohort study, we analyze the experience with genome-wide cfDNA prenatal screening in the autonomous communities of Aragón and Valencia (Spain), between them they have a population of 7 million people. This analysis aims to provide evidence on the clinical utility of expanded NIPT in hospital practice and to assess its potential integration into routine prenatal screening protocols.

## 2. Material and Methods

As this project included two autonomous communities, the recruitment period for pregnant women differed between them. In Aragón, samples were obtained from pregnant patients attended in seven public hospitals belonging to the regional public health system between November 2020 and September 2024, while in Valencia samples were obtained from pregnant patients attended in twenty-one public hospitals belonging also to the regional public health system between January 2023 and December 2024. In general, the test was offered to pregnant women with an intermediate risk (between 1/11 in Aragón or 1/50 in Valencia up to 1/1000 in both places) and a normal ultrasound examination, according to the results of the combined first-trimester screening (cFTS) or second-trimester serum screening (sSTS). It also included women with an intermediate risk estimated from the genetic sonogram, women without prior screening with a gestational age between 19 and 21 weeks, pregnant women aged 40 years or older who were unable to undergo cFTS or sSTS, and patients with a history of live births, spontaneous miscarriages, or voluntary terminations of pregnancy due to trisomy 21, 13, or 18. Additional clinical-obstetric criteria were also considered, such as advanced maternal age, pregnancies with a vanishing twin, or explicit refusal of invasive testing. These inclusion criteria were established according to the Prenatal Screening and Diagnosis Protocol for Fetal Congenital Anomalies of Aragón and according to the Institutional Review Board of Conselleria de Sanidad Universal y Salud Pública (instruction code: 2/2019; approved on 16 April 2019), in force during the study period. Eligible patients were over 18 years of age with a gestational age of at least 10 weeks.

All participants received pre-test counseling and were offered NIPT either in its basic format (focused on the most common trisomies) or in its expanded genome-wide format analyzing all chromosomes. This study was approved by the Research Ethics Committee of Aragón (CEICA) and conducted in accordance with current regulations and the ethical principles set forth in the Declaration of Helsinki. All participants received prior genetic counseling and signed a specific informed consent form.

For cfDNA detection analysis, a peripheral venous blood sample (7–10 mL) was collected via venipuncture in Streck tubes (Streck Corporate, La Vista, NE, USA). Samples were processed using Next-Generation Sequencing (NGS) based technology, VeriSeq™ NIPT Solution v2 (Illumina, Inc., San Diego, CA, USA). Sequencing was performed on the NextSeq™ 550 Dx (Aragón, Spain) or NextSeq™ 500 platform (Valencia, Spain). The data obtained were analyzed using VeriSeq NIPT Assay Software v2 (Illumina, Inc., San Diego, CA, USA), which provides a result of “abnormality detected” (high-risk) or “no abnormality detected” (low-risk) for common trisomies, SCAs, rare autosomal aneuploidies (RAAs), and CNVs >7 Mb. The software also estimated the fetal fraction (FF) during sample analysis, along with the log likelihood ratio (LLR) score used to classify samples as positive or negative based on a predetermined chromosome-specific threshold. Mosaic proportion was also reported as an estimate of the proportion of aneuploid cfDNA detected in the sample.

When a high risk for chromosomal abnormality was identified, patients were promptly contacted to receive thorough post-test counseling. Prenatal diagnostic confirmation was offered through invasive procedures such as chorionic villus sampling or amniocentesis, in accordance with current literature for each specific abnormality. Samples obtained were analyzed using various confirmatory methodologies: conventional karyotyping, quantitative fluorescent PCR (QF-PCR), and array-CGH. In cases of discordance between the NIPT result and the invasive technique, cytogenetic analysis of placental tissue was performed to assess the possible presence of confined placental mosaicism (CPM). This analysis was also carried out in selected cases of pregnancy loss or voluntary termination when viable fetal samples were not available. Regardless of the results of the invasive procedure, pregnancies were classified as “high risk” when a genetic abnormality was confirmed to be present but confined to the placenta.

## 3. Results

Of the 9059 expanded cfDNA tests performed, 132 cases (1.45%) yielded a high-risk result. These included 60 rare autosomal aneuploidies (RAAs), 39 copy number variants (CNVs), 23 sex chromosome aneuploidies (SCAs), and 10 cases of multiple abnormalities—five involving two or more numerical chromosomal abnormalities, and five combining numerical abnormalities with CNVs. For the present analysis, common aneuploidies (trisomies 21, 18, and 13) were not considered. Confirmatory testing was performed in 121 of the 132 cases (91.6%), 116 prenatally and 5 postnatally, with positive results in 24 fetuses and five placental studies (23.9% of the total). Details are shown in Table S1. Among these, 123 were singleton pregnancies, six were twin pregnancies and three involved a vanishing twin. The mean fetal fraction was 9%, ranging from 2% to 24%.

Of the 60 RAAs detected, 59 were trisomies and one was a monosomy. Case distribution is detailed in Table S1. The most frequent RAA was trisomy 7 (20 cases), followed by trisomy 16 (6 cases). Invasive confirmatory testing was performed in 55 cases. Three cases were confirmed (5.5% of total): one trisomy 9, one mosaic trisomy 22, and one mosaic trisomy 14 due to Robertsonian translocation. The trisomy 9 case underwent termination of pregnancy (TOP) due to severe cardiac and cerebral malformations. The mosaic trisomy 22 case also resulted in TOP. The trisomy 14 case occurred in a twin pregnancy, with one fetus showing sonographic evidence of malformations. Invasive testing confirmed mosaic trisomy 14 together with trisomy 13 in the affected fetus, and selective TOP was subsequently performed. Additionally, six placental analyses were conducted, of which three were positive (two trisomy 16 cases and one trisomy 4 case). Overall, confirmation of the NIPT finding was achieved in 6 of the 55 cases (10.9%).

Among the 39 CNVs detected, 23 were large duplications and 16 large deletions. Invasive testing was performed in 36 cases. Five cases—four deletions and one duplication—were confirmed (13.9% of total). The confirmation rate for deletions was 28.6%. One case underwent TOP, and in another, postnatal assessment confirmed certain anomalies (case 30 in Table S1). No information was available for the remaining four cases. Placental analysis was performed in three cases, with one confirmed positive. Confirmation of the NIPT findings was achieved in 6 of the 36 cases (16.6%). Details of these findings are provided in Table S1.

Sex chromosome abnormalities (SCAs) were identified in 23 cases. Invasive testing was performed in 19 cases, with confirmation in 11 (57,9% of the total): three cases of monosomy X (45,X), two of trisomy X (47,XXX), three of Klinefelter syndrome (47,XXY), and three of Jacobs syndrome (47,XYY). In two additional cases, the diagnosis was confirmed through further studies: one postnatal study confirmed one case of monosomy X (45,X), and one placental study confirmed one case of Jacobs syndrome (47,XYY). The confirmation rate was 100% for 47,XYY and 75% for 47,XXY. Thus, genetic studies were conducted in 21 cases, with 13 cases confirmed genetically (61.9% with 6 cases showing discordant results). In only 2 cases were no genetic studies performed.

Among the 10 pregnancies with multiple abnormalities, invasive testing was performed in all, with one (case 101 in Table S1) confirmed positive (10%) and another confirmed in placenta (case 27 in Table S1). Overall, confirmation of the NIPT findings was achieved in 2 of the 10 cases (20%).

Although most cases were singleton pregnancies, there were 9 cases that were not. Among the twin pregnancies, the outcome was confirmed in one of the six cases (16.6%), as previously mentioned (the case of Robertsonian translocation), while in the vanishing twins, it was not confirmed in any of the three.

## 4. Discussion

Currently, there is limited data on the specificity, sensitivity, and positive predictive value (PPV) of expanded NIPT findings for chromosomal abnormalities beyond the common trisomies. This study aims to contribute further evidence regarding its routine use in two autonomous communities in Spain.

Regarding RAAs, the most frequent findings were trisomy 7 and trisomy 16, consistent with previous reports [11]. The PPV after invasive confirmation was 5.5%, with three cases confirmed prenatally; for cases confirmed through placental analysis, the rate was 50% (3/6). According to the meta-analysis by Konya et al. (2025) [12], the RAAs with the highest PPV are trisomies 2, 14, 15, 16, and 22. In our series, confirmed fetal cases included one trisomy 9 (1/2 positive), one trisomy 14 (1/5 positive), and one trisomy 22 (1/3 positive); placental confirmation included two trisomy 16 (2/7 positive) and one trisomy 4 (the only case detected). All three confirmed fetal cases underwent TOP due to significant anomalies. These findings indicate that detection of rare autosomal aneuploidies may have relevant clinical implications. Moreover, when invasive testing yields negative results, many of these cases should not be considered true false positives but rather instances of confined placental mosaicism—particularly involving chromosomes X, Y, and certain autosomes—which may be associated with adverse pregnancy outcomes such as preeclampsia, intrauterine growth restriction, or preterm birth [10,13].

It is well established that the majority of RAAs are confined to the placenta; therefore, confirmation in amniotic fluid is not usually expected, resulting in low PPVs. However, if chorionic villus sampling were performed, a higher proportion of NIPT-positive cases would likely be confirmed, leading to higher PPVs. Since the primary goal of prenatal diagnosis is to determine whether the fetus is affected, amniocentesis remains the recommended confirmatory procedure. Nevertheless, the extension of NIPT to include RAAs raises a potential paradigm shift: in addition to serving as a test for fetal chromosomal abnormalities, it could also function as a screening tool for adverse pregnancy outcomes (e.g., preeclampsia, intrauterine growth restriction, preterm birth). For this purpose, traditional predictive values may not adequately reflect the clinical utility of these findings.

The PPV for CNVs in our cohort was approximately 14%, significantly higher for deletions than for duplications, based on invasive confirmation. This is similar to the rate reported by Raymond et al. (19.1%) [14], but lower than that in other studies (44.1–74.2%) [10,15]. Discrepancies may be due to the limited sample size of our cohort, as well as factors such as maternal CNVs, uterine fibroids, or confined placental mosaicism (CNV was confirmed in one of three placentas analyzed). The five confirmed CNVs corresponded to alterations in critical chromosomal regions with established clinical significance—structural abnormalities that would not have been detected through conventional prenatal screening.

For SCAs, the overall PPV of 58% is consistent with previous studies, with markedly higher values for Y-chromosome–associated aneuploidies (47,XYY and 47,XXY: 75% PPV) compared to X-chromosome abnormalities, particularly Turner syndrome (45,X), which showed a PPV of 38% in our series. These results underscore the clinical utility of cfDNA screening for SCAs and are in line with recent ACMG guidelines, which strongly recommend its use for fetal SCA detection.

It should be mentioned that only for sex chromosome analysis the PPV is relevant. It is worth discussing the potential impact of performing additional invasive procedures when NIPT includes rare autosomal aneuploidies (RAAs) and copy-number variants (CNVs). The rationale for performing fewer invasive techniques is that they carry a small risk of complications and cause a significant increase in initial anxiety before the procedure, although they resolve long-term anxiety by yielding a karyotype result [16]. Moreover, it has been hypothesized that midtrimester amniocentesis may be associated with increased rates of sleep disturbances in children, reflecting potential long-term effects of invasive testing [17].

The heterogeneity of participating hospitals in both regions limited the ability to perform postpartum genetic testing in all cases with positive NIPT and negative amniocentesis results.

The American College of Medical Genetics and Genomics (ACMG) and the International Society for Prenatal Diagnosis (ISPD) acknowledge the promising potential of expanded NIPT to detect rare autosomal aneuploidies (RAAs), copy number variants (CNVs), and other microdeletion syndromes beyond the common aneuploidies [5,18]. The ACMG emphasizes that, despite this promise, there is currently insufficient evidence to support the routine clinical use of NIPT for RAAs or CNVs other than the 22q11.2 deletion, highlighting the need for further research to validate these expanded screening applications and clarify their clinical implications. Similarly, the ISPD points out significant challenges in evaluating expanded NIPT due to variability in technological platforms, diverse population characteristics, and the rarity of some conditions, which makes proper validation in clinical cohorts unfeasible. The ISPD also underscores the complexity of interpreting RAA results, often confounded by confined placental mosaicism, leading to low positive predictive values and emphasizing the necessity of expert counseling and confirmatory diagnostic procedures such as amniocentesis. Both societies concur that current evidence is inadequate to recommend routine screening for these expanded conditions in unselected populations and advocate for additional prospective studies to better assess the clinical performance, utility, and ethical considerations of expanded NIPT. In consonance with these positions, the Spanish Association of Prenatal Diagnosis (AEDP) and the Spanish Association of Human Genetics (AEGH) similarly emphasize, more recently in 2025, the need for more clinical evidence and robust studies before expanded NIPT can be routinely implemented [19].

Overall, these results suggest that, while expanded NIPT may not be universally valid for detecting all types of chromosomal alterations in a screening context, its detection of additional anomalies provides clinically relevant information in a significant proportion of cases. Importantly, identifying such findings does not markedly increase the number of invasive procedures due to the low prevalence of these rarer alterations. However, this opportunistic screening is currently accessible only to patients with an intermediate risk for trisomies 21, 18, or 13, and is not available to the general pregnant population. Further clinical evidence and well-designed research are essential to inform guidelines and optimize the safe and effective integration of expanded NIPT into prenatal care.

## Data Availability

The original contributions presented in this study are included in the article/Supplementary Material. Further inquiries can be directed to the corresponding authors.

## Supplementary Materials

The following supporting information can be downloaded at: https://www.mdpi.com/article/10.3390/genes16111344/s1, Table S1: Overview of RAA, CNVs and SCAs calls by prenatal cfDNA screening.
