# Supplementary material for: Clinical Utility of Opportunistic Genome-Wide cfDNA Prenatal Screening in Intermediate-Risk Pregnancies
Source: Genes (Basel). 2025 Nov 7;16(11):1344. doi: 10.3390/genes16111344 (PMC12652038; doi:10.3390/genes16111344)
Supplement: Supplementary file 1 [file genes-16-01344-s001.zip › genes-3905833-supplementary.pdf]

| Case number | Weeks of gestation | Maternal age | Fetal fraction | Fetus number | Gestation type | Gestation Singleton/Twin | NIPT Result | Confirmatory study | Confirmatory study Result |
|---|---|---|---|---|---|---|---|---|---|
| 32 | 13+2 | 42 | 4% | 1 | natural | Singleton | del(18)(q22.1q23) | Not performed | Not performed |
| 36 | 13+2 | 35 | 5% | 1 | natural | Singleton | del(19)(p13.3q13.43) | amniocentesis (QF-PCR + ArrayCGH)/placenta (karyotype + ArrayCGH) | Amniocentesis negative, placenta del(19) mosaic |
| 26 | 11+4 | 39 | 4% | 1 | natural | Singleton | dup(15)(q14q21.2) + T21 | amniocentesis (QF-PCR + ArrayCGH) | Negative |
| 29 | 13+3 | 39 | 7% | 1 | natural | Singleton | dup(20)(p13p11) | amniocentesis (QF-PCR + ArrayCGH)/placenta (karyotype + ArrayCGH) | Negative |
| 28 | 12+5 | 43 | 6% | 1 | natural | Singleton | dup(5)(p14.3p14.1) | amniocentesis (QF-PCR + ArrayCGH) | Negative |
| 27 | 13+7 | 41 | 4% | 1 | natural | Singleton | T12 + T20 | amniocentesis (QF-PCR + ArrayCGH)/placenta (karyotype + ArrayCGH) | Amniocentesis Negative, placenta T20 mosaic |
| 14 | 18+6 | 28 | 11% | 1 | natural | Singleton | T16 | amniocentesis (QF-PCR + ArrayCGH)/placenta (karyotype + ArrayCGH) | Amniocentesis negative, placenta T16 mosaic |
| 25 | 12+2 | 31 | 9% | 1 | natural | Singleton | T16 | amniocentesis (QF-PCR + ArrayCGH)/placenta (karyotype + ArrayCGH) | Amniocentesis negative, placenta T16 mosaic |
| 60 | 12 | 19 | 10% | 2 | natural | Twin | T2 + T7 + M18 + M20 + M21 + M22 + del(7)(q22.1q32.3) | amniocentesis (QF-PCR + ArrayCGH + karyotype) | Negative |
| 15 | 12+6 | 25 | 6% | 1 | natural | singleton | T7 | amniocentesis (QF-PCR + ArrayCGH)/placenta (karyotype + ArrayCGH) | Negative |
| 24 | 12+4 | 30 | 13% | 1 | natural | Singleton | XXY | amniocentesis (QF-PCR + ArrayCGH) | CONFIRMED |
| 22 | 13+6 | 37 | 14% | 1 | natural | Singleton | XYY | amniocentesis (QF-PCR + ArrayCGH) | CONFIRMED |
| 23 | 11+3 | 40 | 12% | 1 | natural | Singleton | XYY | placenta (karyotype+ ArrayCGH) | CONFIRMED |
| 122 | 12 | 35 | 17% | 1 | natural | Singleton | del(1)(p31.1p13.2) + del(1)(q42.13q44) + M14 | amniocentesis (QF-PCR + ArrayCGH + karyotype) | Negative |
| 119 | 12 | 33 | 11% | 1 | natural | Singleton | del(1)(p36.33p13.2) + T2 + T4 + del(6)(q16.3q24.2) + T8 + T13 | amniocentesis (QF-PCR + ArrayCGH + karyotype) | Negative |
| 90 | 13 | 41 | 4% | 1 | natural | Singleton | del(1)(q25.1q31.1) | amniocentesis (QF-PCR + ArrayCGH + karyotype) | CONFIRMED |
| 30 | 12+2 | 26 | 7% | 1 | natural | Singleton | del(10)(q22.3q23.2) | amniocentesis (QF-PCR + ArrayCGH) | CONFIRMED |
| 96 | 12 | 37 | 8% | 1 | natural | Singleton | del(10)(q26.13q26.3) | amniocentesis (FISH + karyotype) | Negative |
| 73 | 12 | 37 | 11% | 1 | IFV | Singleton | del(13)(q33.1q34) | amniocentesis (QF-PCR + ArrayCGH + karyotype) | Negative |
| 42 | 13 | 39 | 10% | 1 | natural | Singleton | del(14)(q24.3q32.11) | amniocentesis (QF-PCR + ArrayCGH) | Negative |
| 104 | 16 | 38 | 11% | 1 | natural | Singleton | del(15)(q13.3q23) | amniocentesis (QF-PCR + ArrayCGH + karyotype) | Negative |
| 61 | 12 | 32 | 12% | 1 | natural | Singleton | del(17)(q22.1q32.2) | amniocentesis (QF-PCR + ArrayCGH + karyotype) | Negative |
| 69 | 11 | 36 | 15% | 1 | IFV | Singleton | del(18)(q22.1q23) | amniocentesis (QF-PCR + ArrayCGH + karyotype) | CONFIRMED |
| 89 | 12 | 41 | 8% | 1 | natural | Singleton | del(19)(p13.11q13.43) | amniocentesis (ArrayCGH) | Negative |
| 94 | 22 | 24 | 7% | 1 | natural | Singleton | del(22)(q13.2q13.33) | amniocentesis (QF-PCR + ArrayCGH + karyotype) | Negative |
| 108 | 12 | 38 | 5% | 1 | natural | Singleton | del(5)(q14.3q31.3) | amniocentesis (QF-PCR + ArrayCGH + karyotype) | Negative |
| 17 | 12+3 | 38 | 17% | 1 | IFV | singleton | del(7)(q31.32.3) | placenta (karyotype+ ArrayCGH) | Negative |
| 76 | 13 | 31 | 10% | 1 | natural | Singleton | del(7)(q34q36.3) | amniocentesis (QF-PCR + ArrayCGH + karyotype) | CONFIRMED |
| 79 | 11 | 38 | 6% | 1 | natural | Singleton | del(8)(p23.3q11.1) | amniocentesis (QF-PCR + ArrayCGH + karyotype) | Negative |
| 71 | 16 | 38 | 2% | 1 | natural | Singleton | dup(1)(p13.3q32.2) | amniocentesis (QF-PCR + ArrayCGH + karyotype) | Negative |
| 58 | 15 | 34 | 11% | 2 | natural | Twin | dup(1)(p21.3p13.3) | amniocentesis (QF-PCR + ArrayCGH + karyotype) | Negative |
| 13 | 12+3 | 35 | 8% | 1 | natural | Singleton | dup(1)(p32.3p31.3) | amniocentesis (QF-PCR + ArrayCGH) | Negative |
| 33 | 12+6 | 33 | 7% | 1 | natural | Singleton | dup(10)(p12.31p11.22) | amniocentesis (QF-PCR + ArrayCGH) | Negative |
| 113 | 12 | 31 | 4% | 1 | natural | Singleton | dup(11)(q13.5q25) | amniocentesis (QF-PCR + ArrayCGH + karyotype) | Negative |
| 9 | 12+3 | 38 | 10% | 2 | natural | Twin | dup(12)(p13.33 p11.21) | amniocentesis (QF-PCR + ArrayCGH) | Negative |
| 125 | 13 | 44 | 6% | 1 | natural | Singleton | dup(12)(q24.21q24.31) | amniocentesis (QF-PCR + karyotype) | Negative |
| 19 | 12+4 | 36 | 7% | 1 | IFV | Singleton | dup(13)(q21.2q21.33) | amniocentesis (QF-PCR + ArrayCGH) | Negative |
| 43 | 12 | 39 | 10% | 1 | natural | Singleton | dup(18)(p11.32q11.1) | amniocentesis (QF-PCR + ArrayCGH + karyotype) | Negative |
| 7 | 14 | 41 | 6% | 1 | natural | Singleton | dup(18)(p11.32q12.3) | amniocentesis (QF-PCR + ArrayCGH) | Negative |
| 5 | 13+2 | 42 | 11% | 1 | natural | Singleton | dup(19)(p.1311q12) | amniocentesis (QF-PCR + ArrayCGH) | Negative |
| 66 | 12 | 32 | 6% | 1 | natural | Singleton | dup(20)(q11.21q13.12) | amniocentesis (QF-PCR + ArrayCGH + karyotype) | Negative |
| 103 | 15 | 25 | 6% | 1 | natural | Singleton | dup(21)(q11.2q22.13) | amniocentesis (QF-PCR + ArrayCGH + karyotype) | CONFIRMED |
| 130 | 12 | 42 | 5% | 1 | IFV | Singleton | dup(3)(p22.3p21.31) | amniocentesis (QF-PCR + ArrayCGH + karyotype) | Negative |

| | | | | | | | | | |
|---|---|---|---|---|---|---|---|---|---|
| 85 | 13 | 33 | 7% | 1 | natural | Singleton | dup(4)(q28.2q28.3) | amniocentesis (ArrayCGH + karyotype) | Negative |
| 107 | 14 | 38 | 10% | 1 | IFV | Singleton | dup(6)(p22.2q25.1) | amniocentesis (QF-PCR + ArrayCGH + karyotype) | Negative |
| 128 | 13 | 42 | 8% | 2 | natural | Twin | dup(7)(q11.21q33) | amniocentesis (QF-PCR + ArrayCGH) | Negative |
| 109 | 14 | 28 | 8% | 1 | natural | Singleton | dup(7)(q34q36.1) | Not performed | Not performed |
| 3 | 12+2 | 30 | 14% | 1 | natural | Singleton | dup(9)(p24.3p13.1) | amniocentesis (QF-PCR + ArrayCGH) | CONFIRMED |
| 50 | 15 | 40 | 6% | 1 | natural | Singleton | dup(9)(p24.3q13) | ArrayCGH in neonate | Negative |
| 62 | 12 | 41 | 9% | 1 | IFV | Singleton | M13 | amniocentesis (QF-PCR + ArrayCGH + karyotype) | Negative |
| 102 | 13 | 46 | 6% | 1 | IFV | Singleton | T1 | amniocentesis (QF-PCR + ArrayCGH + karyotype) | Negative |
| 124 | 12 | 39 | 3% | 1 | IFV | Singleton | T1 | amniocentesis (QF-PCR + karyotype) | Negative |
| 38 | 18+6 | 38 | 9% | 1 | natural | Singleton | T10 | Not performed | Not performed |
| 63 | 13 | 34 | 5% | 1 | natural | Singleton | T10 | amniocentesis (QF-PCR + ArrayCGH + karyotype) | Negative |
| 64 | 12 | 31 | 13% | 1 | natural | Singleton | T10 | amniocentesis (QF-PCR + ArrayCGH + karyotype) | Negative |
| 72 | 12 | 30 | 10% | 1 | natural | Singleton | T11 | amniocentesis (QF-PCR + ArrayCGH + karyotype) | Negative |
| 4 | 8 | 35 | 9% | 1 | natural | Singleton | T14 | Not performed | Not performed |
| 48 | 13 | 44 | 12% | 1 | IFV | Singleton | T14 | amniocentesis (QF-PCR + ArrayCGH + karyotype) | Negative |
| 84 | 12 | 30 | 11% | 2 | natural | Twin | T14 | amniocentesis (QF-PCR, FISH + ArrayCGH + karyotype) | CONFIRMED in mosaic (T13 + T14) |
| 120 | 13 | 25 | 13% | 1 | natural | Singleton | T14 | amniocentesis (FISH + karyotype) | Negative |
| 131 | 12 | 43 | 15% | 1 | IFV | Singleton | T14 | amniocentesis (QF-PCR + ArrayCGH + karyotype) | Negative |
| 1 | 17 | 39 | 9% | 1 | IFV | Twin | T15 | amniocentesis (QF-PCR + ArrayCGH) | Negative |
| 35 | | 33 | 5% | 0 | natural | Singleton | T15 | Not performed | Not performed |
| 110 | 13 | 39 | 20% | 1 | natural | Vanishing twin | T15 | amniocentesis (FISH + karyotype) | Negative |
| 11 | 11+4 | 36 | 6% | 1 | natural | Singleton | T16 | amniocentesis (QF-PCR + ArrayCGH)/placenta (karyotype + ArrayCGH) | Amniocentesis negative, placenta T16 mosaic |
| 37 | 13+1 | 39 | 24% | 1 | natural | Singleton | T16 | amniocentesis (QF-PCR + ArrayCGH) | Negative |
| 68 | 12 | 41 | 9% | 1 | IFV | Vanishing twin | T16 | Not performed | Not performed |
| 121 | 14 | 29 | 12% | 1 | natural | Singleton | T16 | amniocentesis (QF-PCR + ArrayCGH + karyotype) | Negative |
| 126 | 14 | 38 | 6% | 1 | natural | Singleton | T16 | amniocentesis (QF-PCR + ArrayCGH + karyotype) | Negative |
| 12 | 15+6 | 40 | 5% | 1 | natural | Singleton | T18 + del(1)(q22q24.3) | amniocentesis (QF-PCR + ArrayCGH) | Negative |
| 101 | 14 | 40 | 10% | 1 | natural | Singleton | T18 + XXY | amniocentesis (QF-PCR + ArrayCGH + karyotype) | CONFIRMED |
| 53 | 12 | 32 | 11% | 1 | natural | Singleton | T2 | amniocentesis (QF-PCR + ArrayCGH + karyotype) | Negative |
| 75 | 13 | 40 | 9% | 1 | natural | Singleton | T2 | amniocentesis (QF-PCR + ArrayCGH + karyotype) | Negative |
| 98 | 17 | 30 | 11% | 1 | natural | Singleton | T2 + T6 | amniocentesis (FISH + karyotype) | Negative |
| 21 | 10+3 | 33 | 4% | 1 | natural | Singleton | T20 | Not performed | Not performed |
| 49 | 12 | 39 | 10% | 1 | natural | Singleton | T20 | amniocentesis (QF-PCR + ArrayCGH + karyotype) | Negative |
| 92 | 12 | 39 | 5% | 1 | IFV | Vanishing twin | T20 | amniocentesis (QF-PCR + FISH + karyotype) | Negative |
| 115 | 13 | 41 | 5% | 1 | natural | Singleton | T20 | abortive remains (FISH + karyotype + ArrayCGH) | Negative |
| 52 | 12 | 38 | 13% | 1 | natural | Singleton | T22 | amniocentesis (QF-PCR + ArrayCGH + karyotype) | Negative |
| 80 | 14 | 23 | 22% | 1 | natural | Singleton | T22 | amniocentesis (QF-PCR + ArrayCGH + karyotype) | CONFIRMED in mosaic |
| 97 | 14 | 38 | 3% | 1 | natural | Singleton | T22 | amniocentesis (FISH + karyotype) | Negative |
| 20 | 17+2 | 35 | 9% | 1 | natural | Singleton | T3 | amniocentesis (QF-PCR + ArrayCGH) | Negative |
| 59 | 12 | 38 | 8% | 1 | natural | Singleton | T3 | amniocentesis (FISH + karyotype) | Negative |
| 118 | 14 | 38 | 4% | 1 | natural | Singleton | T3 | amniocentesis (QF-PCR + ArrayCGH + karyotype) | Negative |
| 127 | 12 | 24 | 6% | 1 | natural | Singleton | T3 | amniocentesis (QF-PCR + ArrayCGH + karyotype) | Negative |
| 8 | 13+2 | 35 | 4% | 1 | natural | Singleton | T4 | placenta (karyotype+ ArrayCGH) | CONFIRMED |
| 6 | 12 | 36 | 11% | 1 | natural | Singleton | T7 | amniocentesis (QF-PCR + ArrayCGH) | Negative |
| 16 | 12 | 36 | 5% | 1 | natural | Singleton | T7 | amniocentesis (QF-PCR + ArrayCGH) | Negative |
| 18 | 11+4 | 31 | 8% | 1 | natural | Singleton | T7 | amniocentesis (QF-PCR + ArrayCGH + UDP study) | Negative |
| 44 | 13 | 38 | 6% | 1 | natural | Singleton | T7 | amniocentesis (QF-PCR + ArrayCGH + karyotype) | Negative |
| 45 | 20 | 39 | 6% | 1 | natural | Singleton | T7 | amniocentesis (QF-PCR + ArrayCGH + karyotype) | Negative |
| 46 | 13 | 40 | 8% | 1 | natural | Singleton | T7 | amniocentesis (QF-PCR + ArrayCGH + karyotype) | Negative |
| 56 | 11 | 38 | 10% | 1 | natural | Singleton | T7 | amniocentesis (QF-PCR + ArrayCGH + karyotype) | Negative |
| 57 | 20 | 31 | 7% | 1 | natural | Singleton | T7 | Not performed | Not performed |

| | | | | | | | | | |
|---|---|---|---|---|---|---|---|---|---|
| 74 | 12 | 39 | 22% | 1 | natural | Singleton | T7 | amniocentesis (QF-PCR + ArrayCGH + karyotype + UDP study) | Negative |
| 78 | 20 | 35 | 13% | 1 | natural | Singleton | T7 | amniocentesis (QF-PCR + ArrayCGH + karyotype) | Negative |
| 82 | 16 | 40 | 14% | 1 | natural | Singleton | T7 | amniocentesis (FISH + ArrayCGH + karyotype + UDP study) | Negative |
| 86 | 13 | 31 | 16% | 1 | natural | Singleton | T7 | amniocentesis (QF-PCR + ArrayCGH + karyotype + UDP study) | Negative |
| 87 | 15 | 41 | 12% | 1 | natural | Singleton | T7 | amniocentesis (QF-PCR + ArrayCGH + karyotype) | Negative |
| 88 | 16 | 27 | 10% | 1 | natural | Singleton | T7 | amniocentesis (QF-PCR + ArrayCGH + karyotype + UDP study) | Negative |
| 100 | 20 | 30 | 10% | 1 | natural | Singleton | T7 | amniocentesis (FISH + karyotype) | Negative |
| 111 | 13 | 34 | 6% | 1 | natural | Singleton | T7 | amniocentesis (QF-PCR + ArrayCGH + karyotype) | Negative |
| 123 | 12 | 38 | 18% | 1 | natural | Singleton | T7 | amniocentesis (QF-PCR + karyotype) | Negative |
| 129 | 13 | 32 | 7% | 1 | natural | Singleton | T7 | amniocentesis (QF-PCR + ArrayCGH) | Negative |
| 132 | 15 | 39 | 10% | 1 | IFV | Singleton | T7 | amniocentesis (QF-PCR + ArrayCGH + karyotype) | Negative |
| 70 | 12 | 36 | 9% | 1 | natural | Singleton | T7 + T13 | amniocentesis (QF-PCR + ArrayCGH + karyotype) | Negative |
| 10 | 16 | 39 | 9% | 1 | natural | Singleton | T7 + T8 | amniocentesis (QF-PCR + ArrayCGH) | Negative |
| 93 | 13 | 37 | 8% | 1 | natural | Singleton | T8 | amniocentesis (QF-PCR + FISH + karyotype) | Negative |
| 99 | 12 | 41 | 9% | 1 | natural | Singleton | T8 | amniocentesis (FISH + karyotype) | Negative |
| 114 | 14 | 38 | 8% | 1 | natural | Singleton | T8 | amniocentesis (QF-PCR + karyotype) | Negative |
| 31 | 13+3 | 41 | 12% | 1 | natural | Singleton | T9 | amniocentesis (QF-PCR + ArrayCGH)/placenta (karyotype + ArrayCGH) | Negative |
| 34 | 12+1 | 29 | 6% | 1 | natural | Singleton | T9 | amniocentesis (QF-PCR + ArrayCGH) | CONFIRMED |
| 41 | 14 | 27 | 14% | 1 | natural | Singleton | X0 | amniocentesis (QF-PCR + ArrayCGH) | Negative |
| 77 | 13 | 38 | 9% | 1 | natural | Singleton | X0 | amniocentesis (QF-PCR + ArrayCGH + karyotype) | Negative |
| 81 | 16 | 37 | 4% | 1 | natural | Singleton | X0 | Not performed | Not performed |
| 83 | 14 | 38 | 12% | 1 | natural | Singleton | X0 | amniocentesis (QF-PCR + ArrayCGH + karyotype) | CONFIRMED |
| 91 | 13 | 35 | 7% | 1 | natural | Singleton | X0 | amniocentesis (QF-PCR + karyotype) | Negative |
| 95 | 21 | 25 | 14% | 1 | natural | Singleton | X0 | amniocentesis (QF-PCR + karyotype) | CONFIRMED |
| 106 | 26 | 18 | 13% | 1 | natural | Singleton | X0 | amniocentesis (QF-PCR + karyotype) | CONFIRMED |
| 116 | 12 | 41 | 10% | 1 | IFV | Singleton | X0 | amniocentesis (QF-PCR + karyotype) | Negative |
| 117 | 12 | 29 | 9% | 1 | natural | Singleton | X0 | amniocentesis (QF-PCR + karyotype) | Negative |
| 2 | 14 | 39 | 9% | 1 | natural | Singleton | XXX | Not performed | Not performed |
| 40 | 13 | 42 | 8% | 1 | natural | Singleton | XXX | Karyotype in neonate | Negative |
| 47 | 13 | 37 | 7% | 1 | natural | Singleton | XXX | amniocentesis (QF-PCR + ArrayCGH + karyotype) | CONFIRMED |
| 51 | 12 | 42 | 5% | 1 | natural | Singleton | XXX | amniocentesis (QF-PCR + ArrayCGH + karyotype) | CONFIRMED |
| 39 | 14 | 43 | 6% | 1 | natural | Singleton | XXY | amniocentesis (QF-PCR + ArrayCGH) | CONFIRMED |
| 54 | 12 | 38 | 9% | 1 | natural | Singleton | XXY | amniocentesis (QF-PCR + karyotype) | Negative |
| 55 | 12 | 44 | 7% | 1 | natural | Singleton | XXY | Not performed | Not performed |
| 67 | 12 | 38 | 9% | 1 | natural | Singleton | XXY | amniocentesis (QF-PCR + ArrayCGH + karyotype) | CONFIRMED |
| 105 | 12 | 35 | 6% | 1 | natural | Singleton | XXY | amniocentesis (QF-PCR + karyotype) | CONFIRMED |
| 65 | 15 | 24 | 7% | 1 | natural | Singleton | XYY | amniocentesis (QF-PCR + ArrayCGH + karyotype) | CONFIRMED |
| 112 | 12 | 34 | 8% | 1 | IFV | Singleton | XYY | amniocentesis (QF-PCR + karyotype) | CONFIRMED |
